# Comment on: Exploring the Low Force-High Velocity Domain of the Force–Velocity Relationship in Acyclic Lower-Limb Extensions

**DOI:** 10.1186/s40798-023-00648-7

**Published:** 2023-11-27

**Authors:** Julian Alcazar, Fernando Pareja-Blanco, Ignacio Ara, Luis M. Alegre

**Affiliations:** 1 GENUD Toledo Research Group, Faculty of Sports Sciences, Universidad de Castilla-La Mancha, Toledo, Spain; 2 CIBER On Frailty and Healthy Ageing (CIBERFES), Instituto de Salud Carlos III, Madrid, Spain; 3 Instituto de Investigación Sanitaria de Castilla-La Mancha (IDISCAM), Junta de Comunidades de Castilla-La Mancha (JCCM), Castilla-La Mancha, Spain; 4 Physical Performance and Sports Research Center, Department of Sports and Computer Sciences, Universidad Pablo de Olavide, Seville, Spain; 5 Faculty of Sports Sciences, Department of Sports and Computer Sciences, Universidad Pablo de Olavide, Seville, Spain

To the Editor,

We read with great interest the recent study by Riviere et al. [1] on the force–velocity (F–V) relationship observed in acyclic lower-limb extensions. To give the reader a context, our research group has previously shown that the F–V relationship follows a nonlinear pattern, consisting of a linear portion at moderate-high forces/moderate-low velocities (above ~ 45% of estimated maximum isometric force or *F*_0_) and a curvilinear portion at low forces/high velocities (below ~ 45% of *F*_0_) [2]. In contrast, in their study, Riviere et al. [1] concluded that a linear model is the most appropriate to describe the F–V relationship in acyclic lower-limb extensions, contradicting our findings [2–5]. However, the results provided by Riviere et al. [1] confirm our previous findings indeed, as will be presented below using their reported experimental data.

First, the collection of F–V data requires establishing some inclusion/exclusion criteria following some basic physiological principles in order to avoid the inclusion of submaximal trials [4]: i) force decreases as a function of velocity, and ii) power increases from unloaded conditions to maximum power (*P*_max_), and then decreases until F_0_ is reached. From the average F–V data reported by Riviere et al. [1], at least the second principle was not fulfilled in their experiments (Fig. 1A, B).

**Fig. 1:**
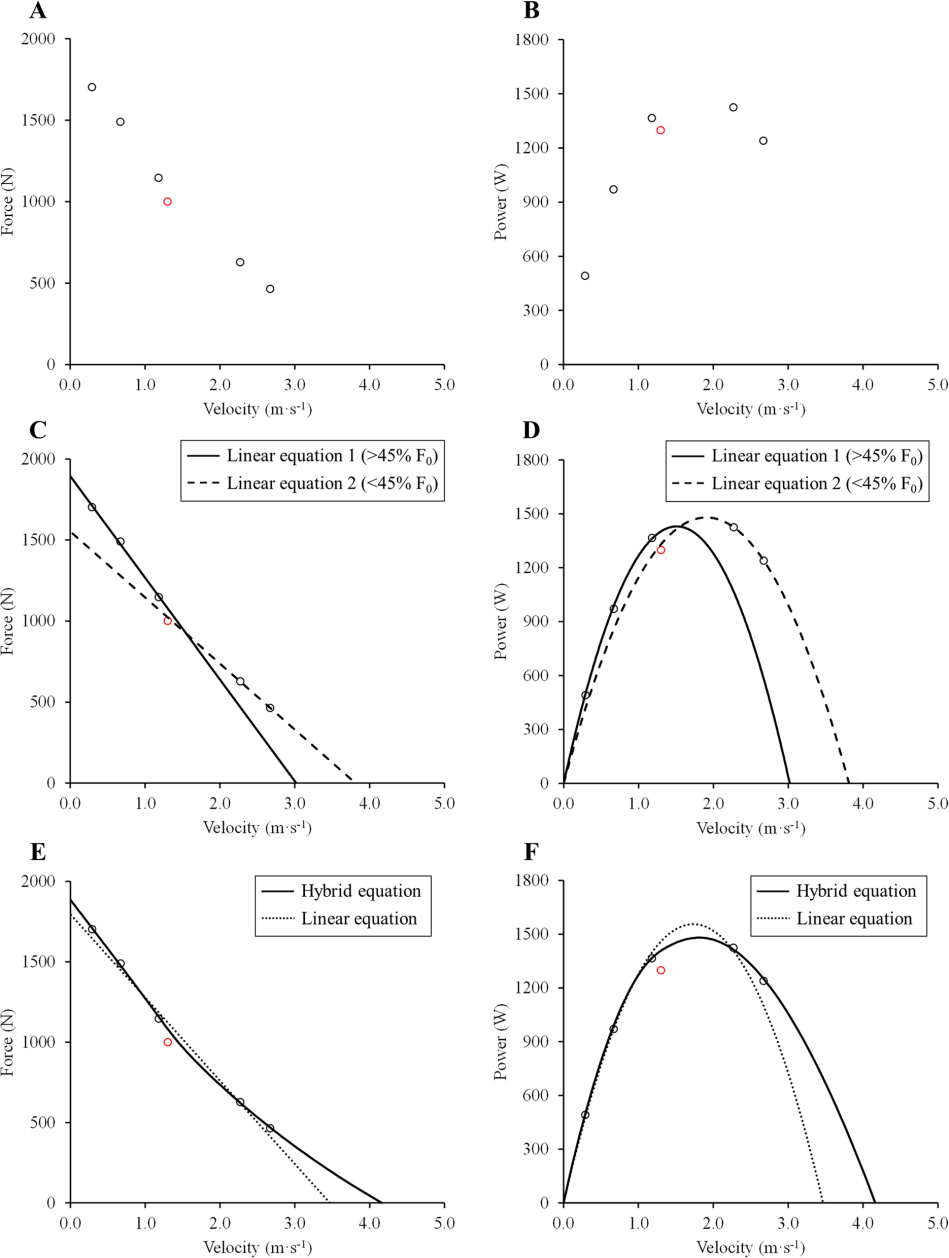
Force–velocity relationship and power-velocity relationship during acyclic lower-limb extensions. Adapted from data from Table 2 in Riviere et al. [1]. **A** and **B** show the reported force–velocity (F–V) and power–velocity (P–V) data by Riviere et al. [1], respectively. Note that one trial (*red open symbol*) does not fulfill the physiological inclusion criteria for the P–V relationship. Only data fulfilling the physiological criteria (*black open symbols*) will be considered for further analyses here (except for the linear equation in **E** and **F**). **C** and **D** show the different results that two linear models applied to different portions of the F–V and P–V relationships provide, respectively. Based on this, the hypothesis that the F–V relationship is linear should be rejected. **E** and **F** show the different results that the hybrid and linear equations yield regarding F–V and P–V data, respectively. As previously reported [4], linear models tend to underestimate F_0_ and V_0_ and overestimate *P*_max_, while showing clear deviations for the actual recorded data, in comparison with the hybrid equation.

On the other hand, the hypothesis that the F–V relationship is linear is relatively simple to test. Due to the nature of linear models, the linear equation obtained from one portion of the F–V relationship (e.g., below 45% *F*_0_) must fit the F–V data collected in a different portion of the F–V relationship (e.g., above 45% *F*_0_). Nonetheless, using data from Riviere et al. [1], the linear equations obtained from different portions of the F–V relationship do not fit the data contained in the other portions of the F–V relationship (Fig. 1C) or P–V relationship (Fig. 1D). Therefore, the F–V relationship is not linear. Of note, this conclusion also reached in previous studies was not simply based on differences in r^2^ and SEE values between equations (as suggested by Riviere et al. [1]), but on significant differences found in the derived parameters (i.e., F_0_, maximal unloaded velocity, P_max_, optimal force and velocity, and F–V slope) [2, 4]. Then, if two different equations provide significantly different outcomes, it seems common sense to conclude that the one that best fits the recorded data is the one that best represents the F–V relationship. In this sense, a hybrid equation combining a linear and a hyperbolic equation provided the best fit to recorded F–V data while yielding physiologically reasonable values (Fig. 1E, F) [4].

Finally, as discussed in our previous studies [4], it is true that the use of linear models may present several advantages that may outweigh its limitations in some situations. Linear models reduce the amount of data to be collected, avoid the need for complex settings to record data at the extremes of the F–V relationship, and provide relevant outcomes related to functional performance in both young [6] and older populations [7]. Nevertheless, the use of linear models needs to be standardized to minimize their limitations. For example, as demonstrated here, two linear equations applied to two different portions of the F–V relationship will provide substantially different results, which complicates the comparison between studies, individuals, or pre-post results using different ranges of F–V data. In this sense, the use of linear models over a standardized range of F–V data (above ~ 45% of *F*_0_ [4]) should be recommended. Moreover, the above-mentioned physiological inclusion criteria should be applied to avoid submaximal attempts to be included in the analyses and contaminate the results.

In conclusion, the F–V relationship in acyclic lower-limb extensions shows a hybrid behavior consisting of a linear and a hyperbolic portion. Hence, the F–V relationship is not linear. However, linear F–V models can still be used in certain contexts based on the goals and available equipment and time, but their limitations need to be acknowledged. When a more valid, detailed, and comprehensive analysis of the F–V and P–V relationships is required, the hybrid equation, which provides physiologically reasonable and functionally relevant outcomes, should be preferred over linear models.

## Data Availability

Not applicable.

## Abbreviations

*F*_0_: Estimated maximal isometric force
F–V: Force–velocity
P–V: Power–velocity
*P*_max_: Maximum muscle power
